# Unexpected Enhancement of High-Cycle Fatigue Property in Hot-Rolled DP600 Steel via Grain Size Tailoring

**DOI:** 10.3390/ma18245658

**Published:** 2025-12-17

**Authors:** Yu Song, Cheng Zhang, Yu-An Chen, Mingyue Yang, Chao Zhang, Bing Lu, Yuhe Huang, Jun Lu, Shuize Wang

**Affiliations:** 1 College of Materials Science and Engineering, Chongqing University, Chongqing 400044, China; 18281219693@163.com; 2 Research Institute for Carbon Neutrality, University of Science and Technology Beijing, Beijing 100083, China; 18727458700@163.com (C.Z.); ymy13592030656@163.com (M.Y.); m202421846@xs.ustb.edu.cn (C.Z.);; 3 Xichang Steel Vanadium Limited Company, Pansteel Group, Xichang 615000, China; 15183432271@163.com

**Keywords:** dual-phase steels, high-cycle fatigue, fatigue crack growth behavior, roughness-induced crack closure effect, crack deflection effect

## Abstract

This work systematically investigates the high-cycle fatigue (HCF) properties and fatigue crack growth (FCG) behavior of hot-rolled dual-phase (DP) steels with comparable tensile strength but distinctly different yield strength (458 MPa for the FG sample and 355 MPa for the CG sample), grain sizes and morphologies. Contrary to the conventional Hall–Petch relationship, the coarse-grained (CG) sample demonstrates superior fatigue performance. This enhancement is reflected in its higher fatigue strength, combined with an elevated FCG threshold and a reduced FCG rate in the Paris regime of FCG behavior. Fracture morphologies and FCG path analyses reveal that this enhanced fatigue resistance attributes to pronounced crack path tortuosity in the CG microstructure. The tortuous crack path enhances roughness-induced crack closure effects in the near-threshold regime while promoting more frequent crack deflection during stable propagation, collectively reducing the effective driving force for crack growth. The experimental evidence confirms that properly designed CG microstructures with appropriate phase distribution can provide superior fatigue resistance in hot-rolled DP steels.

## 1. Introduction

Dual-phase (DP) steels, which feature a microstructure of hard martensite embedded in a soft ferrite matrix, have gained considerable attention in the automotive industry owing to their cost-effectiveness and high-performance [1,2]. This distinctive microstructural configuration endows DP steels with an excellent strength–ductility balance [3], enhanced initial strain hardening [4], and continuous yielding behavior [5]. These attributes make DP steels particularly suitable for sheet-formed components in body-in-white applications, including wheels, beams, and bumpers [6]. In recent years, growing emphasis on sustainable manufacturing has further driven the transition from energy- and carbon-intensive cold-rolled DP steels to more environmentally compatible hot-rolled counterparts [7].

The hot-rolled DP steels as body-in-white components are inevitably exposed to cyclic loading, which makes fatigue resistance important to the design and manufacturing of hot-rolled DP steels [6]. It is well established that the fatigue strength of metallic materials frequently follows a Hall–Petch type relationship with grain size, wherein finer microstructures generally lead to improved fatigue performance [8,9,10]. For instance, Burda et al. refined the grain size of a microalloyed bainite steel from 25 μm to 5 μm via optimized thermo-mechanical-controlled processing (TMCP), reporting that the fine-grained samples exhibited superior fatigue resistance and a lower fatigue crack growth (FCG) rate [11]. In a study on pure aluminum, Wang et al. similarly observed the higher high-cycle fatigue strength in the ultra-fine-grained sample, which can be attributed to the increased fraction of high-angle grain boundaries acting as effective barriers to crack propagation [12]. However, existing research has been primarily focused on single-phase materials, where grain size refinement generally leads to simultaneous enhancement of both tensile and fatigue strength [13]. In such cases, the individual contribution of grain size to fatigue resistance remains conflated with grain-refinement strengthening effects. Consequently, the specific role of grain size in fatigue performance under constant strength conditions has rarely been investigated, particularly in the complex multi-phase systems like DP steels.

In this study, DP steels with comparable tensile strength but contrasting grain sizes were successfully fabricated, and their HCF performance and FCG behavior were systematically compared. Contrary to common perception, the coarse-grained (CG) sample exhibited an unexpected superior fatigue strength and a lower FCG rate. These findings were interpreted in detail through the combined actions of roughness-induced crack closure and crack deflection. These results provide a novel insight into the grain size-dependent fatigue behavior and suggest a new design strategy for developing high-performance hot-rolled DP steels.

## 2. Materials and Methods

### 2.1. Material Preparation

**Figure 1:**
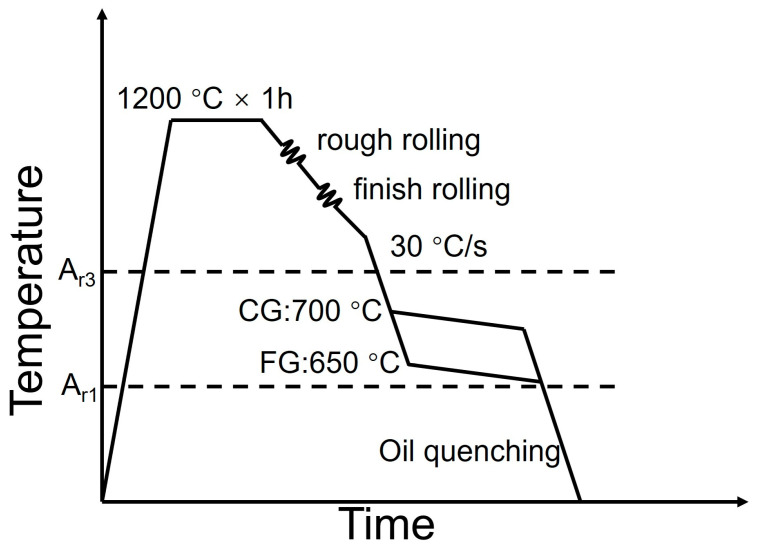
Schematic diagram of thermo-mechanical controlled process for DP steels.

The material used in this study was a commercial hot-rolled DP steel with the following chemical composition (wt.%): Fe-0.05C-0.08Si-0.75Mn-0.58Cr-0.015Nb. To produce two distinct microstructures, coarse-grained (CG) and fine-grained (FG) with comparable tensile strengths, the TMCP parameters were carefully designed, as schematically illustrated in Figure 1. A key design aspect was the use of different initial thicknesses to achieve markedly different total rolling reductions, which is crucial for controlling the prior-austenite grain size. Accordingly, the CG sample was processed from a 50 mm thick intermediate plate, while the FG sample originated from a significantly thicker 220 mm as-cast slab, both being rolled to the same final thickness of 4 mm. For the CG sample, the billet was homogenized at 1200 °C and subsequently rolled via a two-stage hot-rolling process. The plate was then immediately cooled to an intermediate temperature of 700 °C, held for 8 s under air cooling to facilitate ferrite transformation, and finally quenched to room temperature to form martensite. A similar two-stage cooling strategy was employed for the FG sample. However, a lower intermediate cooling temperature of 650 °C was applied to obtain a lower final martensite volume fraction.

### 2.2. Tensile Testing and Microstructure Characterization

**Figure 2:**
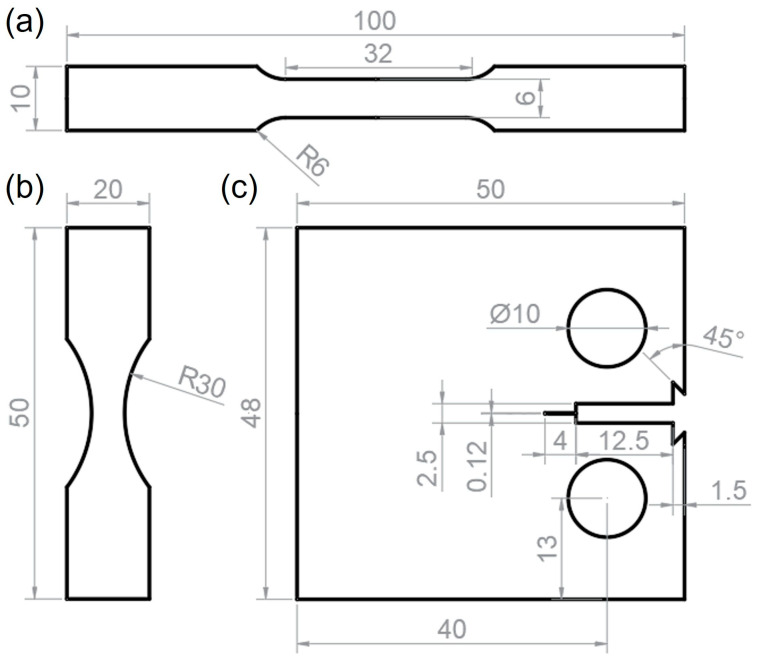
Schematic diagrams of (**a**) tensile specimen, (**b**) high-cycle fatigue test specimen and (**c**) fatigue crack growth specimen.

The uniaxial tensile test for both CG and FG samples was performed on an E45.305 MTS universal testing machine at room temperature, with a constant strain rate of 6.67 × 10^−3^ s^−1^ in accordance with the GB/T 228.1-2021 standard [14]. To ensure statistical reliability, all tensile properties of the CG and FG specimen were averaged from three tests. The detailed geometry of the tensile specimen is illustrated in Figure 2a, and the gauge length is 25 mm.

The microstructural characterization of the DP steels was conducted using field emission scanning electron microscopy (SEM, TESCAN Mira LMS, Brno, Czech Republic). Sample preparation involved sequential grinding to 2000-grit SiC paper, mechanical polishing using 1 μm diamond paste, and final etching with 4% Nital solution. To analyze the fatigue crack growth (FCG) path, electron backscatter diffraction (EBSD) was performed with a step size of 0.2 μm on specimens that underwent vibrational polishing (VibroMet 2) for two hours.

### 2.3. High-Cycle Fatigue and Fatigue Crack Growth Testing

High-cycle fatigue (HCF) tests were performed on an MTS-QBG-100kN testing machine in accordance with the GB/T 3075-2021 standard [15]. The tests were conducted at a frequency of 100 Hz and a stress ratio of R = 0.1 under ambient temperature. The fatigue strength of the DP steels with distinct different grain size was determined using the staircase method. The specimen geometry for the HCF tests is depicted in Figure 2b.

Fatigue crack growth (FCG) rate tests were conducted on an MTS-810 electro-hydraulic servo testing system at a frequency of 10 Hz. Standard compact tension (CT) specimens were machined from the oil-quenched sheets with the notch parallel to the rolling direction (Figure 2c).

## 3. Results and Discussion

### 3.1. Effect of TMCP on Microstructure and Mechanical Property of DP Steels

**Figure 3:**
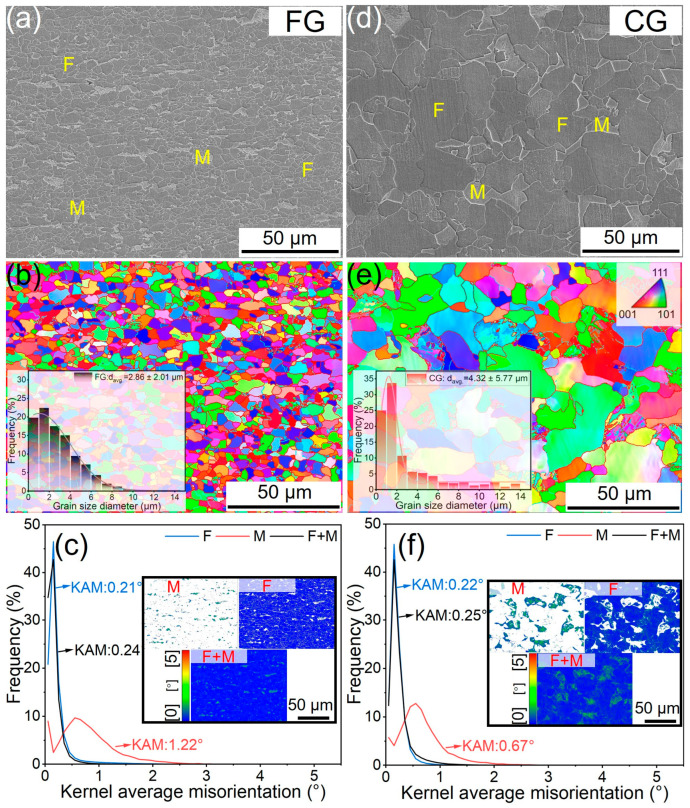
Microstructure characteristics of (**a**–**c**) FG-DP steel and (**d**–**f**) CG-DP steel: (**a**,**d**) SEM images, (**b**,**e**) IPF-Z maps and (**c**,**f**) KAM curves. Note that F and M refer to ferrite and martensite, respectively.

Figure 3a–f present a comparative analysis of the microstructures in the FG- and CG-DP steels. The FG-sample displays a uniform distribution of fine martensite elongated along the rolling direction within the ferrite matrix. In contrast, the CG-sample exhibits coarser martensite, randomly distributed and predominantly located at ferrite grain boundaries. EBSD analysis (Figure 3b,e) confirms that the higher rolling reduction and lower intermediate temperature promoted a finer prior-austenite structure in the FG steel, leading to refined ferrite grains (~2.86 μm) and a higher ferrite fraction (~94%). Conversely, the CG-DP steel, processed with a higher intermediate temperature, developed coarser ferrite grains (~4.32 μm, even up to 10 μm) and a lower ferrite content (~86%).

The observed refinement of ferrite grains with higher rolling reduction can be interpreted through established thermodynamic approaches. This process is governed by the Gibbs free energy change for the austenite to ferrite transformation, expressed as [16,17]:

(1) $$\Delta G_{\gamma \to \alpha }=-V\Delta G_{chem.}+A_{\gamma /\alpha }\sigma_{\gamma /\alpha }+V\Delta G_{V}-V\Delta G_{D}-A_{\gamma }\sigma_{\gamma /\gamma }$$

where ΔG_γ→α_ is the total Gibbs free energy change, ΔG_chem._ is the chemical driving force, A_γ/α_ and A_γ_ represent the interfacial areas of ferrite–austenite and austenite–austenite boundaries, σ_γ/α_ and σ_γ/γ_ are the corresponding interface energy, which are treated as constants, ΔG_V_ is the volume strain energy, ΔG_D_ is the stored deformation energies. The formulation of Equation (1) relies on classical assumptions for phase transformation kinetics, including constant interfacial energies and isotropic growth of the ferrite phase. An increase in rolling reduction raises both ΔG_D_ and A_γ_, thereby enhancing the overall driving force (ΔG_γ→α_) for ferrite transformation. Since austenite grain boundaries serve as preferential nucleation sites for ferrite, the refined prior-austenite microstructure resulting from severe plastic deformation during hot rolling effectively promotes the nucleation rate of ferrite grains [18].

Additionally, Figure 3b,e also reveal that the martensite blocks, which are the key structural units controlling martensite ductility, are notably larger in the CG sample due to its larger prior austenite grain size [7,19,20]. As shown in Figure 3c,f, the average KAM value of M in the CG-DP sample is significantly lower (~0.67°) than that in the FG-DP sample (~1.22°). However, the average KAM values for the F and F+M are comparable between the two conditions, with the FG sample measuring 0.21° and 0.24°, and the CG sample measuring 0.22° and 0.25°, respectively. The hardness of the martensite can be estimated using following Equation [21]:

(2) $$C_{M}=\frac{C_{T}-C_{F}V_{F}}{V_{M}}$$

(3) $$Hv_{M}\approx 903.6C_{M}+45.7$$

where C_T_, C_M_ and C_F_ are the carbon content of total composition, martensite and ferrite, respectively; V_M_ and V_F_ are the corresponding volume fraction; Hv_M_ is the martensite hardness. Based on these relations, the martensite hardness is calculated as 316 Hv for the CG-sample and 651 Hv for the FG-sample. Hence, it is anticipated that the martensite in the CG-DP steel has higher ductility, which will alleviate stress concentration during plastic deformation due to the lower hardness difference between ferrite and martensite [22].

**Figure 4:**
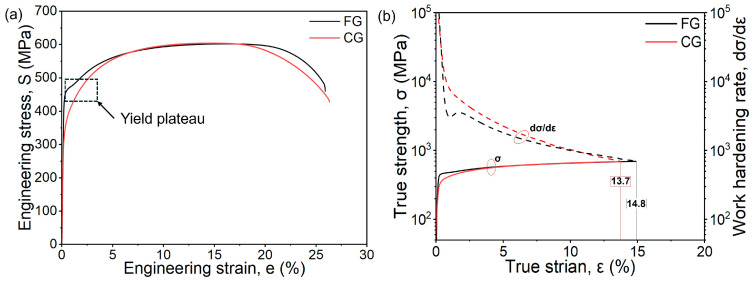
(**a**) Engineering strain versus engineering stress curve and (**b**) true strain and true stress curve.

Despite the significant microstructural differences between the FG and CG samples, their tensile properties are comparable. As summarized in Table 1 and shown in Figure 4a,b, both samples exhibit similar tensile strengths and total elongations. Specifically, the tensile strength and total elongation of FG-DP steel are 601 MPa and 25.4%, while the values for CG-sample are 604 MPa and 25.9%. The true stress–strain curves and the corresponding work hardening rate curves were derived from the engineering stress–strain data, using the following relationships [23]:

(4) $$\sigma =S\left(1+e\right)$$

(5) $$\varepsilon =\ln\left(1+e\right)$$

where S and e are the engineering stress and strain, and σ and ε represent the true stress and true strain, respectively. With increasing strain, the work hardening rates of both the CG and FG samples gradually decrease. According to the Considère criterion [24], the uniform elongation is 13.7% for the CG sample and 14.8% for the FG sample. At larger strains, the true stress–strain curves of the two specimens nearly coincide, indicating similar mechanical responses during uniform elongation. This discontinuous yielding behavior is primarily attributed to the pinning effect of Cottrell atmospheres, and similar phenomena have also been reported in the literature [25].

These mechanical responses can be explained by microstructural features and established models. The pronounced yield plateau in the FG steel is associated with its high ferrite fraction. Previous studies have shown that when the martensite content is below about 10%, the deformation behavior is primarily governed by the ferrite phase [22,26]. In addition, the overall tensile strength σ_b_ of DP steels can be estimated by the rule of mixtures:

(6) $$\sigma_{b}=\sigma_{F}V_{F}+\sigma_{M}V_{M}$$

where σ_F_ and σ_M_ are the strength of ferrite and martensite, respectively. For CG sample, the higher martensite fraction provides greater phase transformation strengthening. However, the grain refinement strengthening in CG sample is lower due to coarser ferrite grains. The interplay of these opposing factors results in a tensile strength similar to that of the FG sample.

### 3.2. HCF Test and FCG Behavior of DP Steels

**Figure 5:**
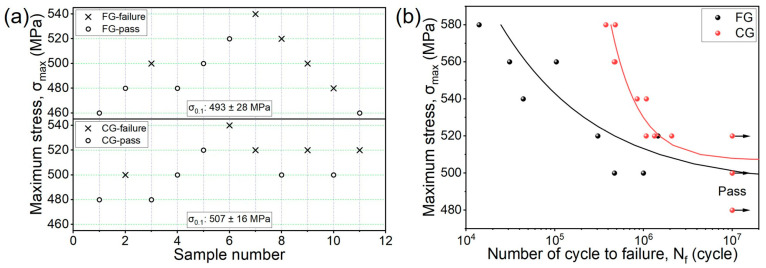
(**a**) Up and down diagrams and (**b**) S-N curves of samples.

The high-cycle fatigue limits of the FG- and CG-DP steels, determined using the staircase method, are measured as 493 MPa and 507 MPa, respectively (Figure 5a). The relationship between the maximum stress (σ_max_) and the number of cycles to failure (N_f_) is fitted using the classical Weibull equation [27]:

(7) $$FG:\log\left(N_{f}\right)=9.294-2.528\log\left(\sigma_{\max}-507\right)$$

(8) $$CG:\log\left(N_{f}\right)=6.992-0.730\log\left(\sigma_{\max}-493\right)$$

Notably, the CG-DP steel exhibits a higher fatigue strength than the FG steel, despite their comparable tensile properties (Figure 5b). This result appears to contradict the general trend that fatigue strength typically increases with grain refinement analogous to the Hall–Petch effect [12]. Moreover, it is observed that the fatigue life of the CG sample was nearly an order of magnitude longer than that of the FG sample below 580 MPa.

**Figure 6:**
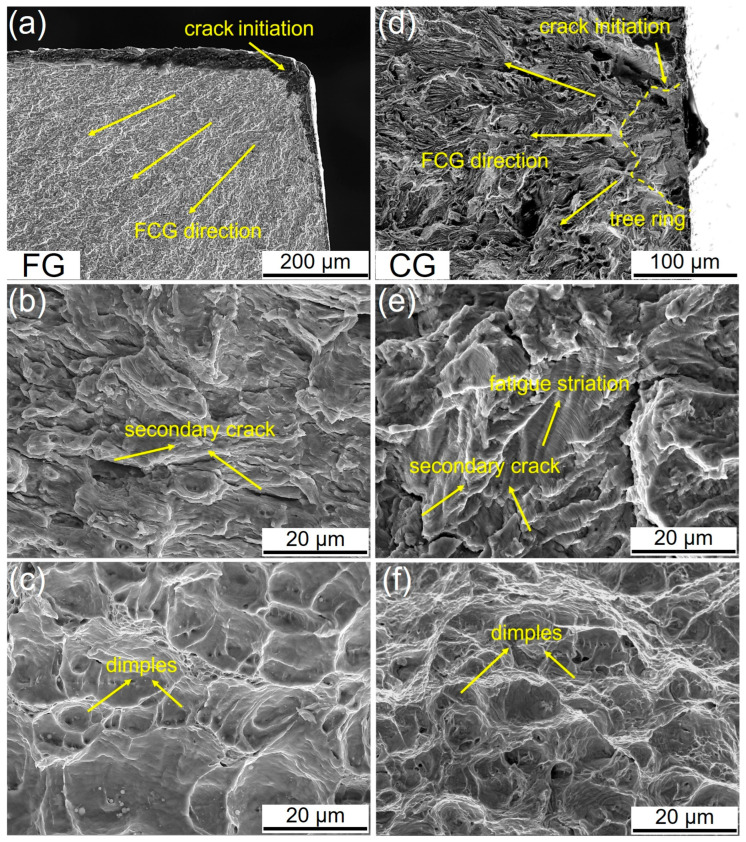
Typical SEM morphology taken from fatigue failure specimen at σ_max_ = 520 MPa for (**a**–**c**) FG-sample and (**d**–**f**) CG-sample.

To further investigate the influence of microstructures on HCF properties, typical fracture morphologies of the FG- and CG-DP steels tested at the same stress amplitude (~520 MPa) are shown in Figure 6. Based on morphological characteristics, the fracture surface can be divided into three distinct regions: the fatigue crack initiation zone, the crack propagation zone, and the final rapid fracture zone. For low-strength steels (<1000 MPa), fatigue cracks generally initiate at the specimen surface and propagate inward in a fan-shaped pattern [13]. In the FG sample, the fatigue cracks originate from geometric discontinuity on the surface (Figure 6a). In contrast, the fatigue cracks initiate at micro-defect induced by cyclic loading in the CG-sample (Figure 6d). Furthermore, a distinct “tree-ring” feature is observed in the crack propagation zone of CG-sample, indicating significant crack growth retardation due to interactions with microstructural barriers [28]. Figure 6b,e show magnified images of the crack propagation zone. Both steels exhibit similar morphologies in this region characterized by secondary cracks and fatigue striations. Meanwhile, Figure 6c,f present the final fracture zone, where both samples show typical ductile fracture covered by dimples. However, the CG sample displays more equiaxed and deeper dimples than the FG sample, indicating superior toughness during FCG process.

As shown in S-N curves in Figure 5b, the difference in fatigue life between the CG and FG samples is minor at low stress amplitudes but increases significantly with increasing stress amplitude. This behavior is attributed to a transition in the dominant fatigue mechanism from crack initiation at low stresses to crack propagation at high stresses. Under stress-controlled HCF conditions, crack initiation typically constitutes a large portion of total life, even up to 80% in some predictions [29,30]. However, with increasing stress level, the accumulated plastic deformation per cycle reduces the crack incubation period and accelerates crack growth, and the crack propagation rate is therefore the dominant factor controlling fatigue life.

**Figure 7:**
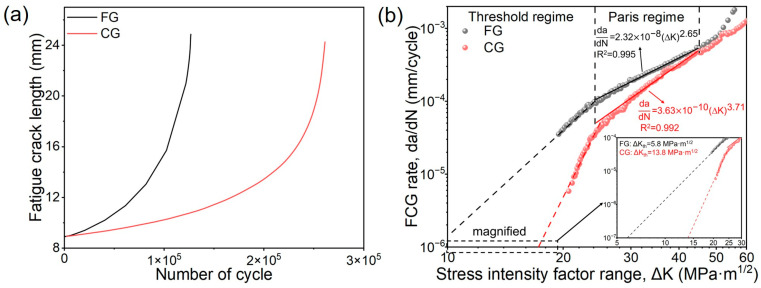
(**a**) Crack length versus number of cycles and (**b**) fatigue crack growth rate curves of FG- and CG-DP steels.

To further examine the effect of grain size on FCG behavior, FCG rate tests were conducted on both samples. As shown in Figure 7a, under the same number of cycles, the crack length in the CG sample is shorter than that in the FG sample, indicating higher crack resistance of the former. The relationship between the FCG rate (da/dN) and the stress intensity factor range (ΔK) is presented in Figure 7b. The da/dN-ΔK curve can be divided into three stages according to its slope, the near-threshold regime, the Paris regime, and the rapid propagation regime. The FCG threshold (ΔK_th_), obtained by extrapolating the curve to da/dN = 10^−7^ mm/cycle, is 5.8 MPa·m^1/2^ for the FG steel and 13.8 MPa·m^1/2^ for the CG sample [20]. It is important to note that ΔK_th_ obtained through extrapolation methods are only suitable for relative comparisons, and the specific values are not precise. In the Paris regime, the FCG behavior follows the Paris equation:

(9) $$\frac{da}{dN}=C{\left(\Delta K\right)}^{m}$$

where C and m are the FCG coefficient and exponent, respectively. The C and m are 2.32 × 10^−8^ and 2.65 for FG-sample, while the values for CG counterpart are 3.63 × 10^−10^ and 3.71. The specific fitting parameters are summarized in Table 2. It is worth noting that as ΔK increases, the difference in FCG rates between the two steels gradually decreases, suggesting that the influence of microstructure on crack propagation weakens at higher stress intensity range.

**Figure 8:**
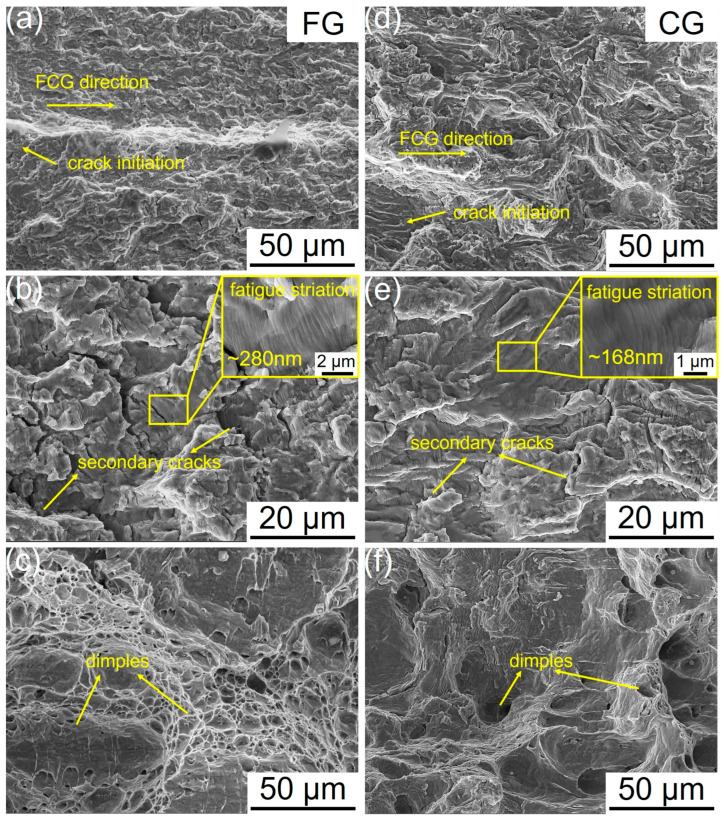
Typical fatigue fracture surface morphology of FG- and CG-DP steels. (**a**,**d**) Fatigue crack initiation at notch, (**b**,**e**) crack stable-propagation regime (ΔK ≈ 30 MPa·m^1/2^) and (**c**,**f**) crack instable-growth region.

Figure 8 shows the fracture surface morphologies of the FG- and CG-DP steels after FCG testing. Both steels exhibit similar fracture characteristics. In the crack initiation zone, distinct initiation sites and steps formed by the convergence of different fracture planes are visible (Figure 8a,d). In the stable crack growth region, secondary cracks and typical fatigue striations are the predominant FCG features. The spacing between fatigue striations corresponds to the crack advance per cyclic loading [31]. Measurements from high-magnification images reveal an average striation spacing of 280 nm in the FG steel and 168 nm in the CG steel, indicating superior crack growth resistance in the CG sample under the same stress intensity conditions (ΔK ≈ 30 MPa·m^1/2^), which is consistent with the FCG rate curves in Figure 7 (Figure 8b,e). With further crack propagation, fatigue striations are gradually replaced by tough dimples, characteristic of a typical ductile fracture morphology (Figure 8c,f).

It is noteworthy that the CG-DP steel exhibits a higher ΔK_th_ and a lower FCG rate than the FG-DP steel, indicating superior fatigue crack propagation resistance despite its coarser microstructure. The underlying mechanisms for this phenomenon will be discussed in detail in the following section.

### 3.3. Effect of Grain Size on FCG Thresholds of FG- and CG-DP Steels

FCG threshold, as a microstructure-sensitive parameter, has a negative relationship with tensile strength and positive relationship with grain size [32,33]. The enhancement of ΔK_th_ in CG steel can be attributed to the higher roughness induced crack closure effect, which can be qualitatively evaluated with the cyclic plastic deformation size (Δr_p_):

(10) $$\Delta r_{p}=\frac{1}{3\pi }{\left(\frac{\Delta K}{2\sigma_{y}}\right)}^{2}$$

where ΔK is the stress intensity factor range in the ahead of crack tip, σ_y_ is the yield stress [34]. It is widely believed that a transition from slow growth to Paris regime will occur as the Δr_p_ becomes comparable to the characteristic microstructural size in the material, such as grain size, block size in the martensite [35]. Hence, the FCG behavior in the threshold regime is influenced by single ferrite grain.

**Figure 9:**
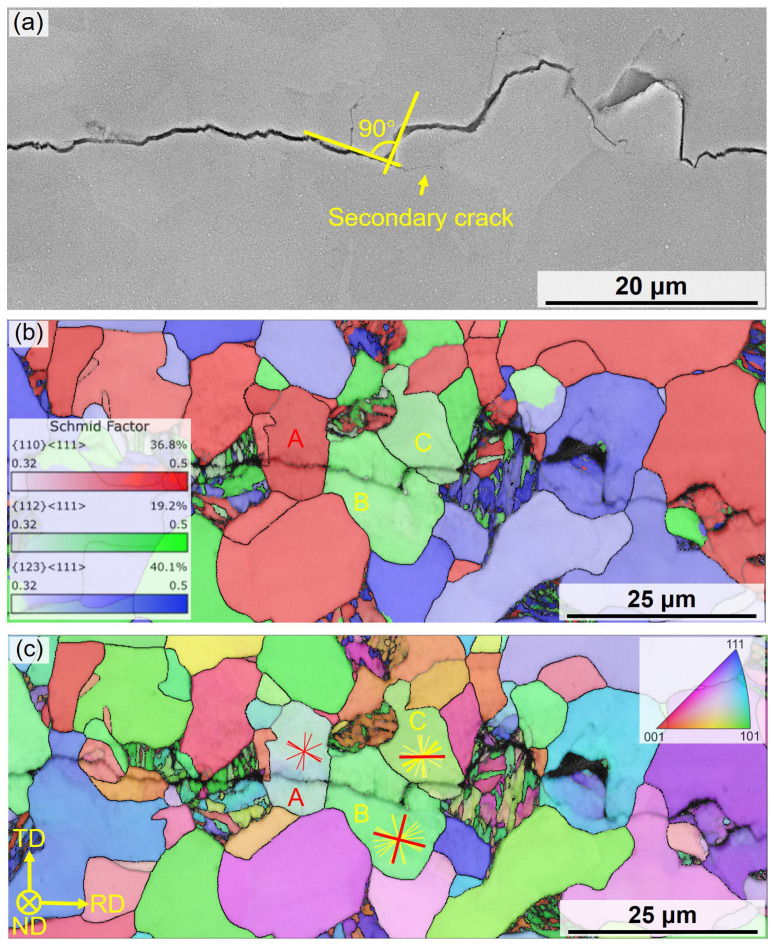
Typical crack propagation SEM and EBSD morphology of CG-DP steel near the threshold regime: (**a**) SEM image, (**b**) Schmid factor map and (**c**) IPF map.

A distinct crack deflection is observed at Grain B on Figure 9. Soft ferrite is generally considered to provide limited resistance to crack propagation, and transgranular cracks within a single ferrite grain rarely undergo significant deflection [36]. EBSD analysis was used to interpret this phenomenon. Figure 9b shows the Schmid factor distribution maps for the three active slip systems in BCC ferrite, which are {110}<111>, {112}<111>, and {123}<111>, under cyclic loading along the transverse direction (TD). The results indicate that plastic deformation in Grain A is primarily controlled by the {110}<111> slip system, whereas in Grains B and C, it is dominated by {112}<111>. By combining the IPF map in Figure 9c with trace analysis of the corresponding slip planes, the crack deflection angle is found to align with the theoretical angle between active slip planes. This suggests that crack deflection in Grain B results mainly from cross-slip of dislocations. Similar results are found in Refs. [37,38].

In the threshold regime, plastic strain is typically localized within single ferrite grain, with limited strain transfer to adjacent grains. In CG-DP steel, dislocations possess a relatively long mean free path and are generally confined to a single grain. When dislocation motion along a primary slip system is hindered, cross-slip facilitates crack deflection. Conversely, in FG-DP steel, Δr_p_ usually extends across multiple grains. The higher density of crack initiation sites and shorter mean free path for dislocation motion promote slip transmission to neighboring grains, often leading to intergranular crack propagation. However, the fine grain size restricts the extent of crack deflection.

**Figure 10:**
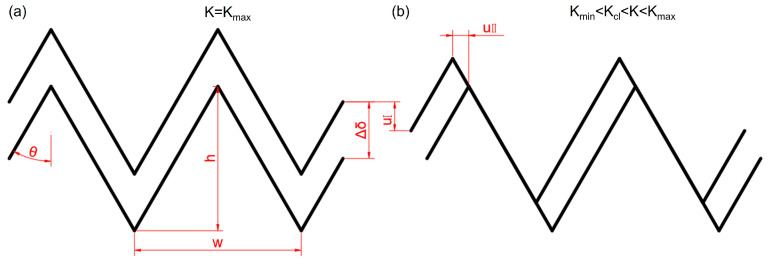
Schematical illustrations of roughness-induced crack closure effect. (**a**) The crack is fully opened and (**b**) the crack is fully closed.

As illustrated in Figure 10a, the crack opening displacement reaches its maximum (Δδ) when K attains K_max_. In this analysis, the actual FCG path is idealized as a triangular waveform, defined by a deflection angle (θ), a peak-to-valley height (h), and a spatial wavelength (w). Since fatigue crack growth in the near-threshold regime is primarily governed by dislocation slip, a geometric mismatch develops between the upper and lower fracture surfaces. This mismatch occurs not only in the loading direction (displacement u_I_) but also perpendicular to it (displacement u_II_), as shown schematically in Figure 10a,b. During the unloading process of cyclic loading, these non-conforming surfaces come into premature contact, reducing the effective stress intensity factor (K_eff._ = K_max_ − K_cl_) at the crack tip. This shielding mechanism is referred to as roughness-induced crack closure [39].

The extent of roughness-induced crack closure can be quantified using the following expression [36]:

(11) $$\frac{K_{cl}}{K_{\max}}\approx \sqrt{\frac{2\gamma \chi }{1+2\gamma \chi }}$$

Here, γ denotes the nondimensional fracture surface roughness factor, which can be determined from the crack path profile [26], and χ represents the ratio of u_I_ to u_II_, indicating the degree of mismatch between the upper and lower fracture surfaces. With increasing crack deflection, the ratio K_cl_/K_max_ also rises, resulting in a lower effective stress intensity factor K_eff._. Consequently, the CG-DP steel exhibits a stronger roughness-induced crack closure effect and a higher Kth than the FG-DP steel. It should be noted that the roughness-induced crack closure effect gradually diminishes as the stress intensity factor at the crack tip increases. When K_min_ > K_cl_, this effect no longer significantly influences the crack growth rate. However, as shown in Figure 7b, a distinct difference in the crack growth rate between the two steels persists even in the Paris regime, where ΔK is relatively high. This indicates that other mechanisms have become dominant in controlling the FCG behavior in this regime.

### 3.4. Effect of Grain Size on FCG Behavior of FG- and CG-DP Steels in Paris Regime

**Figure 11:**
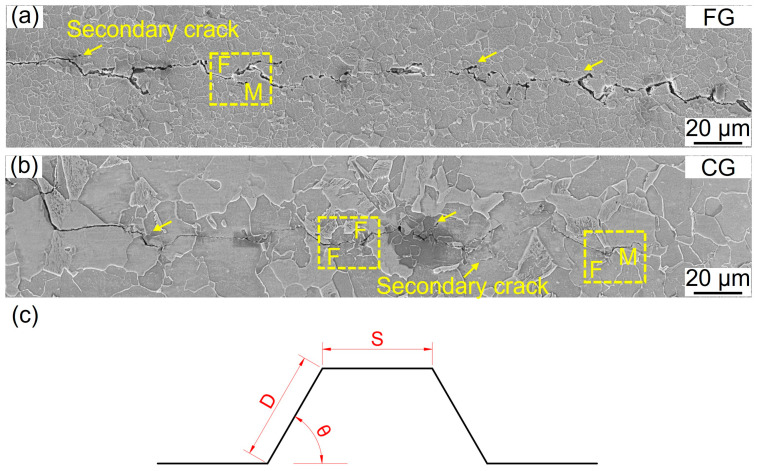
FCG path of FG- and CG-DP steels in the Paris regime. (**a**,**b**) SEM images; (**c**) schematical illustrations for FCG path.

**Figure 12:**
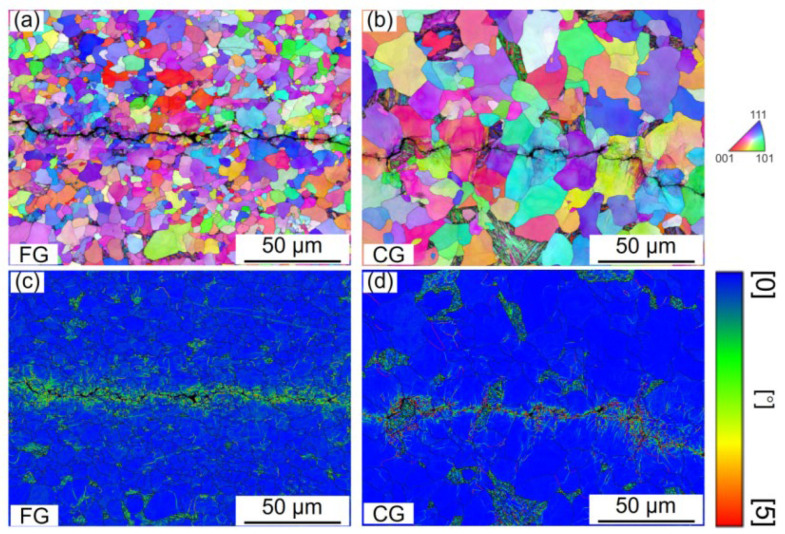
FCG path of FG- and CG-DP steels in the Paris regime. (**a**,**b**) IPF-Z maps and (**c**,**d**) KAM maps.

To further elucidate the governing mechanism of FCG in the Paris regime, the FCG paths of both samples were examined using SEM and EBSD, as shown in Figure 11. Both the FG- and CG-DP steels exhibit a serrated, zigzag crack propagation morphology, accompanied by secondary cracks adjacent to the main crack path (Figure 11a,b), yellow arrows). As shown in Table 3, the CG sample exhibits a higher average deflection angle (44.54° vs. 37.89° for FG) and a greater actual-to-projected crack length ratio (1.15 vs. 1.03 for FG). However, the CG-DP steel demonstrates a more pronounced degree of crack deflection, which is attributed to its higher martensite volume fraction. When the crack encounters hard martensite, the significant hardness difference between ferrite and martensite promotes crack deflection along the phase boundaries, causing the crack to bypass the martensite. This observation is consistent with previous studies, which have established that the extent of crack deflection increases with the martensite content [40]. Figure 12a,b further exhibit the IPF-Z map of FCG path. In the CG steel, crack deflection is markedly pronounced and occurs more frequently than in the FG steel, which is driven by its higher martensite content and coarser martensite size. Furthermore, the corresponding KAM maps adjacent to the FCG path are presented in Figure 12c,d. The KAM analysis confirms that plastic deformation is highly localized, confined to only one or two grains surrounding the crack path, while the majority of the material remains in the elastic strain regime under high-cycle fatigue loading.

Based on the simplified two-dimensional model proposed by Suresh et al., the FCG driving force, considering solely the crack deflection effect, can be quantitatively described as [38]:

(12) $$\Delta K=\left\{\frac{D\;\cos^{2}\left(\frac{\theta }{2}\right)+S}{D+S}\right\}\Delta K_{L}$$

where D represents the actual crack propagation distance along the deflection direction, θ denotes the deflection angle, S signifies the equivalent linear propagation distance, and ΔK_L_ indicates the driving force corresponding to the crack propagating the same distance S without deflection. Therefore, based on the average crack deflection angle and the actual-to-projected crack length ratio in Table 3, ΔK was estimated using Equation (12). The results show that ΔK = 0.93ΔK_L_ for the CG specimen and ΔK = 0.98ΔK_L_ for the FG specimen. This indicates that the value of ΔK in the FG specimen approaches that of a straight-path scenario without deflection. It is worth noting that quantifying the extent of crack deflection over the entire propagation region remains challenging. This is primarily due to the non-uniform crack deflection along the propagation path, with the degree of deflection gradually decreasing as K increases. This also explains why the crack growth rates of the CG and FG specimens become nearly identical in the Paris regime. Due to the coarse martensite size, the CG-DP steel has higher D value, leading to lower ΔK. Furthermore, when considering only the effect of crack deflection, the rate of crack extension can be expressed as follows [41]:

(13) $$\frac{da}{dN}=\left(\frac{D\cos\theta +S}{D+S}\right){\left(\frac{da}{dN}\right)}_{L}$$

where ${\left(\frac{\mathrm{da}}{\mathrm{dN}}\right)}_{L}$ indicates the FCG rate corresponding to the crack propagating the same distance S without deflection. Hence, even in the high K level, the CG-sample still exhibits a lower FCG rate due to the higher crack tortuosity.

### 3.5. FCG Behavior Model of FG- and CG-DP Steels

**Figure 13:**
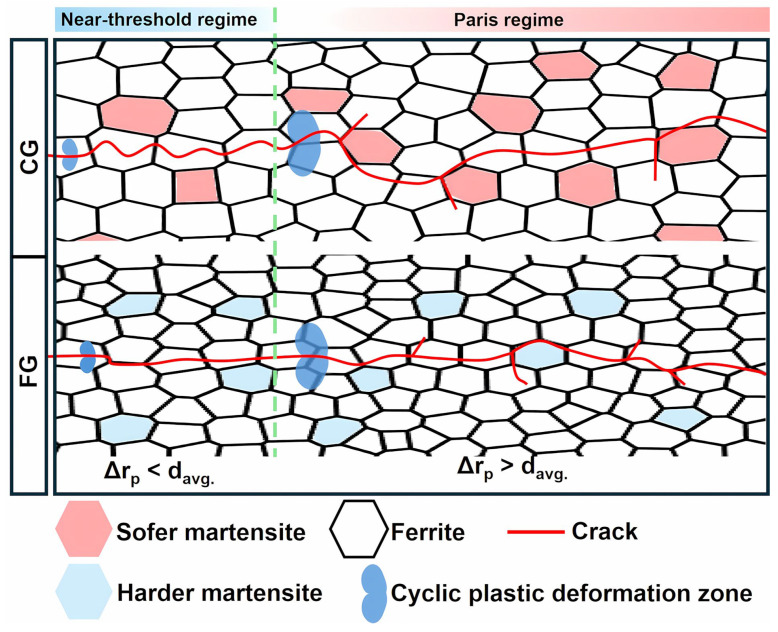
Schematical illustrations of FCG behavior of FG- and CG-DP steels. The green line marks the boundary between the near-threshold regime and Paris regime.

Based on the analysis and calculation in Section 3.3 and Section 3.4, a model describing the FCG behavior of FG- and CG-DP steels is established, as schematically illustrated in Figure 13. In the early stage of crack growth, Δr_p_ at the crack tip is smaller than average grain size (d_avg._). As a result, the FCG behavior is primarily governed by dislocation slip within the ferrite matrix. In the CG-sample, the mean free path for dislocation motion is relatively long, facilitating stress relaxation through cross-slip, which promotes crack deflection. In contrast, in the FG-sample, the shorter dislocation mean free path leads to pronounced dislocation pile-up. The resulting stress concentration is readily transmitted to adjacent grains, favoring intergranular crack propagation and resulting in less crack deflection. Consequently, the roughness-induced crack closure effect is weaker, leading to a lower ΔK_th_ in the FG steel.

With increasing K, Δr_p_ exceeds d_avg._, and the crack growth enters the Paris regime, where the microstructure of both phases collectively influences FCG behavior. Although the contribution of roughness-induced crack closure diminishes in this regime, the CG steel with higher martensite content and larger martensite size exhibits more frequent and pronounced crack deflection. This enhanced tortuosity reduces the effective driving force for crack propagation, resulting in a lower FCG rate in CG steel.

Thus, until the onset of the accelerated growth region, the CG-DP steel consistently maintains a lower FCG rate compared to the FG-DP steel. This model provides an alternative perspective for designing high-performance fatigue-resistant DP steels.

## 4. Conclusions

Based on a carefully designed TMCP route, DP steels with comparable tensile properties but distinctly different grain sizes were successfully produced. The main findings are summarized as follows:

(1) The FG-DP steel has finer ferrite grains (~2 μm) and a lower martensite volume fraction (~6%), while the CG-DP steel show coarser ferrite grains (~4 μm) and a higher martensite content (~14%). These samples exhibit nearly identical tensile strength due to synergistic effect of grain refinement strengthening and hard martensite strengthening.
(2) The CG steel exhibits a slightly higher fatigue strength of 507 MPa, compared to 493 MPa for the FG steel. More notably, under higher stress amplitudes, the fatigue life of the CG sample became nearly an order of magnitude longer than that of the FG sample, indicating a significant microstructural influence on FCG behavior.
(3) In the near-threshold regime, Δr_p_ is smaller than d_avg._. Under such conditions, crack growth is primarily governed by dislocation slip. In the CG-sample, the longer mean free path for dislocation motion facilitates cross-slip within the grain interior, leading to frequent crack deflection. This results in enhanced roughness-induced crack closure, which contributes to a higher ΔK_th_ in the CG steel. In contrast, the restricted dislocation motion in the FG sample results in a relatively straight crack path with limited deflection, thereby reducing the crack closure effect and resulting in a lower ΔK_th_.
(4) In the Paris regime, Δr_p_ exceeds d_avg._, and crack propagation becomes influenced by the DP microstructure. The CG steel, with its higher martensite content and larger martensite size, exhibits more frequent and pronounced crack deflection. This increased crack path tortuosity effectively reduces the local driving force (ΔK), leading to a lower crack growth rate in the CG sample compared to the FG counterpart.

It should be noted that the present study is primarily focused on the HCF properties and FCG behavior under a fixed stress ratio (R = 0.1). The potential effects of the characterized microstructures on other critical mechanical properties, such as impact toughness and low-cycle fatigue performance, were not explored, which represents a limitation of this work. Furthermore, the sensitivity of the fatigue response to different R-ratios remains an open question. Investigations into these aspects would constitute important avenues for future research, and their outcomes are essential for providing comprehensive material selection guidelines for automotive components subjected to complex cyclic loading.

## Figures and Tables

**Table 1:** Mechanical property of FG- and CG-DP steels.

Samples	Yield Strength (MPa)	Tensile Strength (MPa)	Total Elongation (%)	Martensite Volume Fraction (%)
FG	458	601	25.4	6
CG	355	604	25.9	14

**Table 2:** Fitting results of Paris regime.

Samples	Coefficient of FCG Rate, C	Exponent of FCG Rate, m	Correlation Coefficient
FG	2.32 × 10^−8^ ± 1.45 × 10^−9^	2.65 ± 0.02	0.995
CG	3.63 × 10^−10^ ± 4.48 × 10^−11^	3.63 ± 0.03	0.992

**Table 3:** The characteristic parameters of FCG paths.

Samples	Average Deflection Angle	Actual-to-Projected Crack Length Ratio
FG	37.89 ± 15.71°	1.03 ± 0.01
CG	44.54 ± 15.45°	1.15 ± 0.01

## Data Availability

The original contributions presented in this study are included in the article. Further inquiries can be directed to the corresponding authors.
